# A cytoskeleton structure revealed by super-resolution fluorescence imaging in inner ear hair cells

**DOI:** 10.1038/s41421-018-0076-4

**Published:** 2019-02-19

**Authors:** Jieyu Qi, Yan Liu, Cenfeng Chu, Xin Chen, Weijie Zhu, Yilai Shu, Shuijin He, Renjie Chai, Guisheng Zhong

**Affiliations:** 1 iHuman Institute, ShanghaiTech University, Shanghai, China; 2 Key Laboratory for Developmental Genes and Human Disease, Ministry of Education, Institute of Life Sciences, Southeast University, Nanjing, China; 3 Institute for Stem Cell and Regeneration, Chinese Academy of Science, Beijing, China; 4 Co-innovation Center of Neuroregeneration, Jiangsu Key Laboratory of Neuroregeneration, Nantong University, Nantong, China; 5 School of Life Science and Technology, ShanghaiTech University, Shanghai, China; 6 RENT Institute and Otorhinolaryngology, Department Affiated Eye and ENT Hospital, State Key Library of Medical Neurobiology, Fudan University, Shanghai, China; 7 NHC Key Laboratory of Hearing Medicine, Fudan University, Shanghai, China; 8 Research Institute of Otolaryngology, No.321 Zhongshan Road, Nanjing, China

**Keywords:** Actin, Cell biology

Dear Editor,

F-actin is expressed in almost all cells, and plays a variety of important roles^1,2^. In the cuticular plate of hair cells, they are thought to be critical in mammalian hearing by holding the stereocilia rootlets in place and providing the rigidity and support necessary for auditory transduction^3,4^. Due to the high spatial resolution and molecular specificity, the F-actin structure in several types of cells has been recently revealed by super-resolution fluorescence imaging^5,6^. However, how F-actin performs their function in inner ear hair cells remains elusive, partly due to an incomplete understanding of their structure. Here we applied structured illumination microscopy (SIM) imaging to study the sub-diffraction-limit structures of F-actin in the cuticular plate of rodents in order to better understand their function in the development of hearing.

A dense F-actin meshwork has been reported to exist underneath the apical plasma membrane in the cuticular region of hair cells^3,7,8^. Using SIM imaging, we observed ring-like structures of F-actin with a small point cluster at the center, outlining the edges of both inner hair cell (IHC) and outer hair cell (OHC) stereocilia rootlets (Fig. 1a), consistent with the previous EM reconstructed results that F-actin forms two rows of ring-like structure wrapping around the stereocilia rootlets in OHCs^7^. Importantly, a previously unseen fan-shaped meshwork was observed in the OHCs (Fig. 1). The fan-shaped structure was observed in different regions of the cochlea (Fig. 1b), implying a common organization of F-actin in the cuticular plate. Similar to OHCs, ring-like structures of F-actin were observed outlining the edges of IHC stereocilia rootlets (Supplementary Fig. S1a). The small point cluster at the center of each F-actin ring is directly corresponding to individual rootlet (Supplementary Fig. S1b). To investigate how the ring and fan-shaped structure F-actin is organized in the cuticular plate, we visualized the structure of the other actin-associated proteins, such as α-actinin. α-actinin cross-links F-actin bundles and often plays a crucial role to organize F-actin structure. Earlier studies showed the expression of α-actinin in the cuticular plate^9^, and likely it plays a role in organizing the structure of F-actin in the cuticular plate. Here, we found that α-actinin was specifically expressed in the cuticular plate and did not observe the ring or fan-shape like structure of α-actinin with super-resolution imaging experiments (Supplementary Fig. S2).

**Fig. 1:**
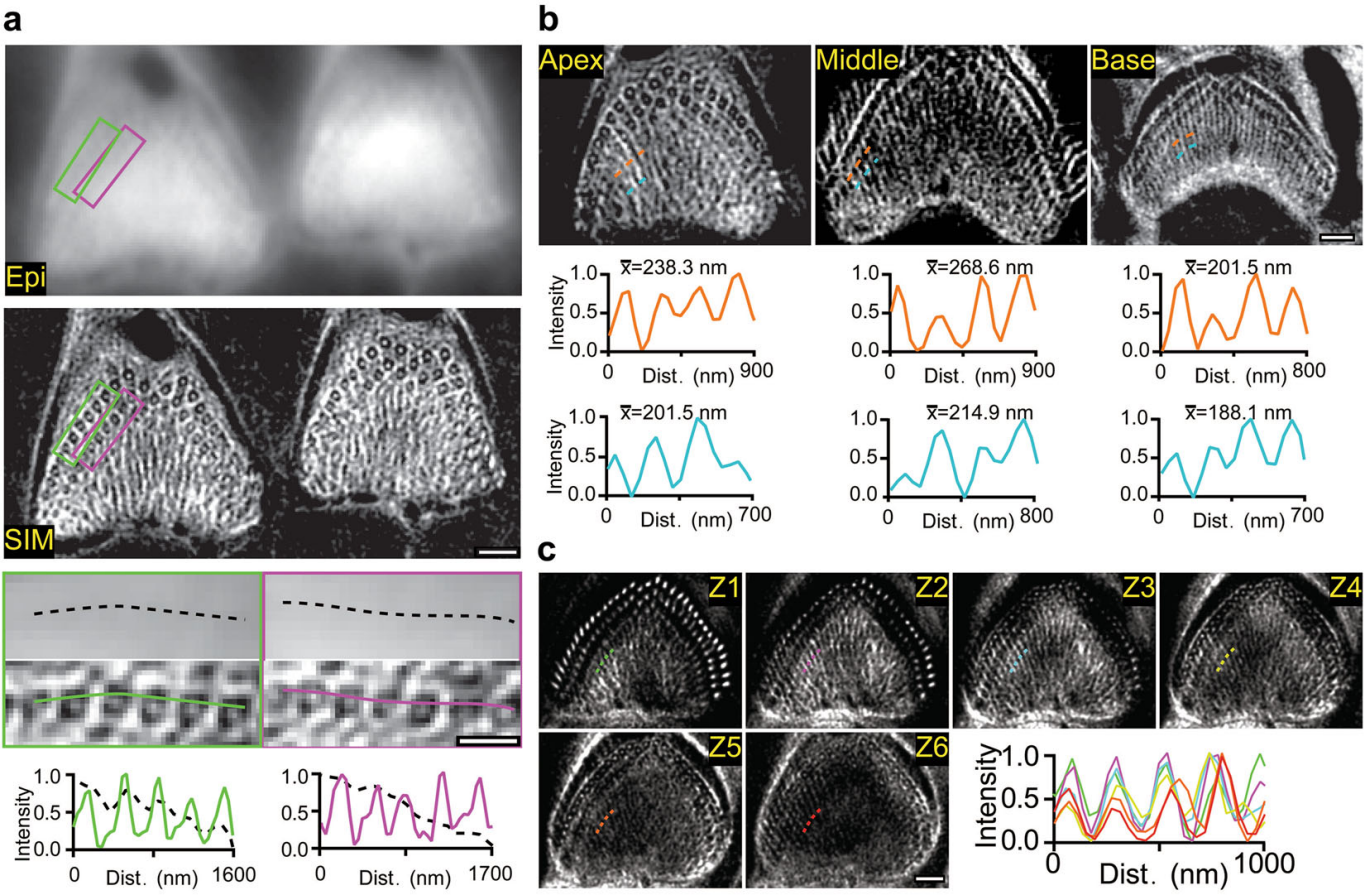
Structure of F-actin in the cuticular plate. **a** Representative conventional epifluorescence vs. SIM fluorescence images of F-actin in the cuticular plates of OHCs from P12 mice (*n* = 5 mice). Magnifications of the boxed region and intensity profiles from the corresponding lines are shown. **b** Representative SIM images of F-actin in the cuticular plates from P12 mice at the apex (*n* = 3 mice), middle (*n* = 3 mice) and base (*n* = 3 mice) region of the cochlea. Intensity profiles along dashed lines are shown. x̅ shows the average distance between two adjacent peaks. **c** Representative serial optical sections (interval = 0.125 μm) of SIM images starting from the apical surface towards the deep region of the cuticular plate of OHC (P12, *n* = 5 mice) along the *Z*-axis. Intensity profiles along the dotted lines are shown. Scale bars: 1 μm in upper panel **a**, 500 nm in lower panel **a**, 1 μm in **b**, **c**

The regular structural pattern of F-actin prompted us to ask whether this structure remained in three-dimensional space of the cuticular plate. We performed three-dimensional SIM experiments. SIM images showed that the F-actin rings and fan meshwork were aligned at different depths that extended more than 750 nm into the cuticular plate of OHCs (Fig. 1c, Supplementary Fig. S3, Supplementary Movies S1-6). Such three-dimensional organized regular pattern of the F-actin cytoskeleton may strength its elasticity, increase the stability of cuticular plate and thus is suitable to hold stereocilia in place after deflections with sounds. F-actin rings were also observed in rat OHCs and IHCs (Supplementary Fig. S4), suggesting that this framework for the spatial patterning of the stereocilia rootlets in the cuticular plate may reflect a common phenomenon in mammalian HCs.

To test whether these structures existed in the early stages of hair cell development, we observed the F-actin structures at different postnatal developmental stages. F-actin concentrated in the cuticular plate of OHCs as early as 0 days after birth (P0), and interestingly no F-actin fan-shaped structure was detected (Supplementary Fig. S5a). Fan-shaped meshwork of F-actin were observed in the cuticular plate of OHCs as early as P7, and remained visible at P30 (Supplementary Fig. S5a-c, Supplementary Movies S1-6). Next, we found that F-actin rings were formed in early development and remained until the mature stage (Supplementary Movies S1-6). Notably, the average diameter of stereocilia rootlets is larger in P0 than that in mature hair cell (Supplementary Fig. S6). Earlier EM experiments elegantly reconstructed the ring-like structure of F-actin in the cuticular plate^10^, and here we extended the earlier results to observe the F-actin bundle which corresponds to each stereocilium and quantitated the properties of the F-actin bundle in the cuticular plate during development with SIM experiments (Supplementary Fig. S6). Our new results suggest that F-actin in rootlets may undergo a developmental structural organization and become more compact and thus support the stereocilia deflection during sound detection. SIM imaging demonstrated that F-actin rings and fan-shaped meshwork appeared in OHCs from the first week after birth and remained to the mature stage. Functionally, the structures of F-actin in the cuticular plate of the hearing impaired *Atoh1-Brg1*^−*/*−^ mice^11^ with OHC morphology disrupted (Supplementary Fig. S7a) showed that, as expected, their rings were severely disrupted in the OHCs, and characteristic fan-shaped meshwork of F-actin had entirely disappeared (Supplementary Fig. S7). Given our findings, it seems likely that the absence of the F-actin rings and the F-actin fan-shaped meshwork would lead to the disruption of the stereocilia rootlets, changing their spatial pattern in the cuticular plate and eventually leading to the disappearance of stereocilia in OHC (Supplementary Fig. S7b). These results support that the organization of the cuticular cytoskeleton is associated with the proper development of stereocilia and thus contributing to the hearing loss in these mice.

In summary, this study has characterized the F-actin nanoscale structures in the cuticular plate with super-resolution imaging method. Our results demonstrate that F-actin forms ring-like structures corresponding to each stereocilium and develops a previously unknown fan-shaped network. Such spatial organization of F-actin in cuticular plate may play a critical role in hearing function.

## Electronic supplementary material

Supplementary Information

Supplementary Movie S1

Supplementary Movie S2

Supplementary Movie S3

Supplementary Movie S4

Supplementary Movie S5

Supplementary Movie S6

## Electronic supplementary material

**Supplementary Information** accompanies the paper at (10.1038/s41421-018-0076-4).
