# Supplementary material for: A cytoskeleton structure revealed by super-resolution fluorescence imaging in inner ear hair cells
Source: Cell Discov. 2019 Feb 19;5:12. doi: 10.1038/s41421-018-0076-4 (PMC6379372; doi:10.1038/s41421-018-0076-4)
Supplement: Supplementary file 1 — Supplementary Information [file 41421_2018_76_MOESM1_ESM.pdf]

Supplementary Information

Materials and Methods

Animals

C57BL/6 mice, Sprague-Dawley rat, *Atoh1-Cre* mice, and *Brg1*$^{flox/flox}$ of both sexes were used. *Brg1*$^{flox/flox}$ mice were mated with *Atoh1-cre* mice to spatially eliminate brg1in inner ear hair cells (HCs). The day of birth was counted from postnatal day 0 (P0). Genotyping primers for *Brg1*$^{flox/flox}$ and *Atoh1-Cre* mice are described as followings: *Brg1*$^{flox/flox}$, forward 5'-TCT CAT GCA CAG AGG TCC TG-3', reverse 5'-TAG CCC CTT GAA AGT GAT CC-3'; *Aoth1*-WT, forward 5'-TGA CGC CAC AGC CAC CTG CTA-3', reverse 5'-GGA CAG CTT CTT GTC GTT GTTG-3'; *Aoth1-Cre*, forward 5'-GCG CAG CGC CTT CAG CAAC-3', reverse 5'-GCC CAA ATG TTG CTG GAT AGT-3'. Animals were housed under a 12 h light/dark cycle at a room temperature of 22 ± 1°C with food and water available ad libitum. All experiments were approved by the Institutional Animal Care and Use Committee of ShanghaiTech University and Southeast University, China.

Immunohistochemistry

Mice were sacrificed with an overdose of pentobarbital sodium (100 mg/kg body weight, i.p.), and the temporal bone was rapidly dissected out under the stereoscope in cold PBS (pH 7.2). To obtain whole-mount preparations of the organ of Corti from mice prior to P7, the cochlear spiral dwelling at the temporal bone was microdissected and adhered to a microscope slide cover glass (thickness 0.17 mm, diameter 10 mm) coated with Cell Tak (BD Bioscience). Cochleae were fixed for 1 hour in 4% paraformaldehyde (in PBS, pH 7.2) at room temperature. For cochleae from mice older than P7, the temporal bone was fixed in 4% paraformaldehyde (in PBS, pH 7.2) for 2 hours at room temperature before being cut into pieces after 0.5 mM EDTA (pH 8.0) treatment. After complete washing with 0.01M PBS, samples were immersed in blocking solution containing 10% donkey serum and 0.3% Triton-X100 in PBS (pH 7.2) for 1–2 h at room temperature. To label F-actin in whole-mount rodent cochlear preparations, phalloidin conjugated with fluorescent dyes (Alexa Fluor® 488, Thermo Fisher Cat# A12379; ATTO 488, ATTO-TEC Cat# AD488-82) was used. After the staining procedures, all samples were carefully examined under a conventional confocal microscope (Zeiss LSM710), and only those samples with high specificity and preferable signal to noise ratio were selected for further super-resolution imaging.

Structured Illumination Microscopy (SIM) Imaging

SIM images of organ of Corti samples were acquired with a GE DeltaVision OMX microscope equipped with a 488-nm laser (coherent), an oil immersion 60x / NA 1.42 objective (PlanApo N, Olympus), and a scientific CMOS (sCMOS) camera (Acquisition pixel size: 82 nm at 60x objective; PCO edge). 3D-SIM mode was used for image acquisition, and 15 images per color were taken with 3 angles and 5 phases for each Z-section. Images were taken in fast 286 MHz mode, with 0.125 μm section spacing. To obtain images with minimized spherical aberration and optimized illumination contrast, immersion oil with different refractive indexes was systematically optimized for each sample. The raw images were reconstructed by the OMX SI reconstruction tool available in softWoRx (GE). Channel-specific OTF files, channel-specific K0 angles, Wiener filter constant (0.001), and bias offset (65) were used during the reconstruction

process.

Scanning Electron Microscopy

Cochlear specimens from P14 and P30 mice were fixed in 2.5% glutaraldehyde (in PBS, pH 7.2) at 4°C overnight and then cut into pieces and post-fixed into osmic acid (Sigma-Aldrich) for 1–2 h. Specimens then were washed and dehydrated in an ethanol gradient and critical-point dried with liquid CO_2 (CPD300, Leica). After electrically conductive coating, the cochlear tissues were observed in random fields using a field-emission SEM (Quanta 250, FEI).

Image Processing and Analysis

All Images were exported from softWoRx (GE) and further processed by Fiji software (National Institutes of Health). The brightness and contrast of the entire images were linearly adjusted. For quantitatively analyzing the distribution pattern of F-actin, lines across the structures were drawn and the intensity profiles were measured by Fiji. Autocorrelation analyses were perform in Matlab (MathWorks, Inc). All of the intensity and autocorrelation data were plotted using GraphPad Prism (GraphPad Software, Inc.), and all the figure layouts were prepared in Illustrator (Adobe Systems, Inc.).

Statistical Analysis

Statistical analyses were conducted by Excel (Microsoft) and GraphPad Prism 6.0 software. Student's *t*-test were used to determine the statistical significance. *$p < 0.05$ was considered significant. All replicate numbers (number of mice, or number of OHCs and IHCs analyzed) are specifically indicated in the figure legends.

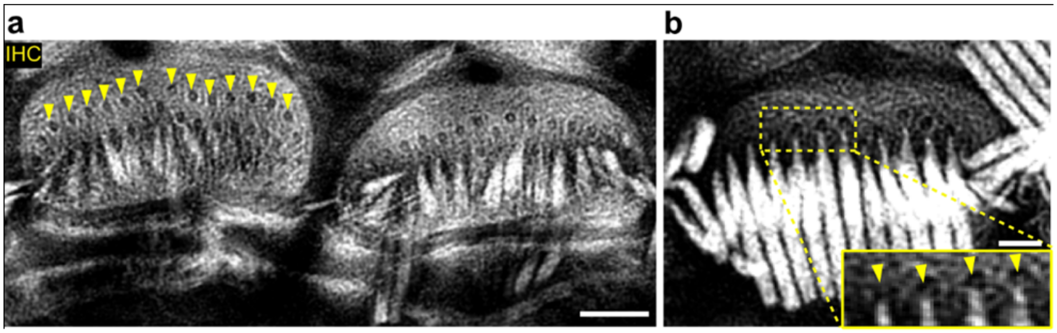

Supplementary Figure S1. Structures of F-actin in IHC.

a, Representative SIM images of F-actin in the cuticular plates from IHCs (n=3 mice). **b,** Representative SIM image (n=3 mice) of F-actin in the apical surface of IHC and magnification of the yellow boxed region. Each yellow arrowhead indicates a point cluster that corresponds single stereocilium. Scale bars: 1 μm.

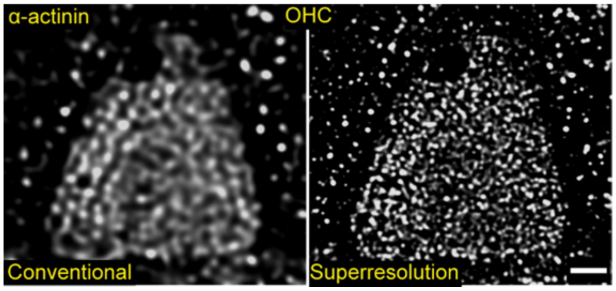

Supplementary Figure S2. Structures of α-actinin in OHC.

Representative confocal and superresolution images of α-actinin in the cuticular plates from OHCs (n=3 mice). Scale bar: 1 μm.

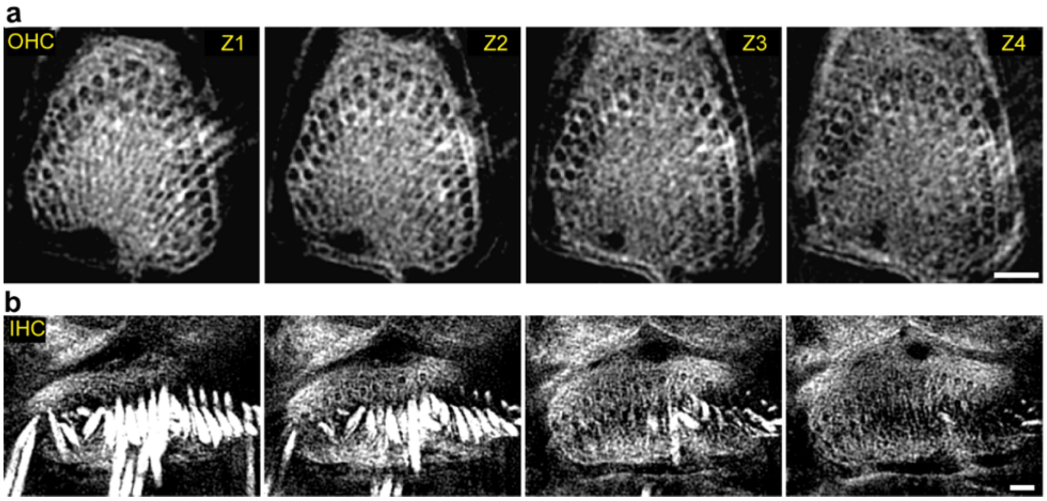

Supplementary Figure S3. 3D Structures of F-actin both in OHC and IHC.

a, Four successive serial optical sections (interval = 0.125 μm) of SIM images of F-actin inside the cuticular plate region in OHC (n=3 mice) from P21 mice. **b,** Same as a except that F-actin structure in IHC (n=3 mice) was analyzed. Scale bars: 1 μm.

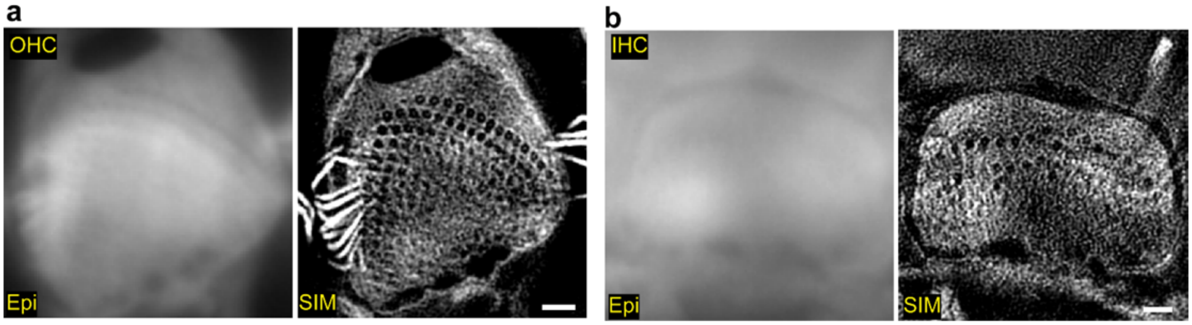

Supplementary Figure S4. F-actin structure in the cuticular plate of rat HCs.

a, Conventional epifluorescence and SIM images of F-actin in the cuticular plate of a rat OHC at P21 (n=3 rats). **b,** Conventional epifluorescence and SIM images of F-actin in the cuticular plate of a rat IHC at P21 (n=3 rats). Scale bars: 1 µm.

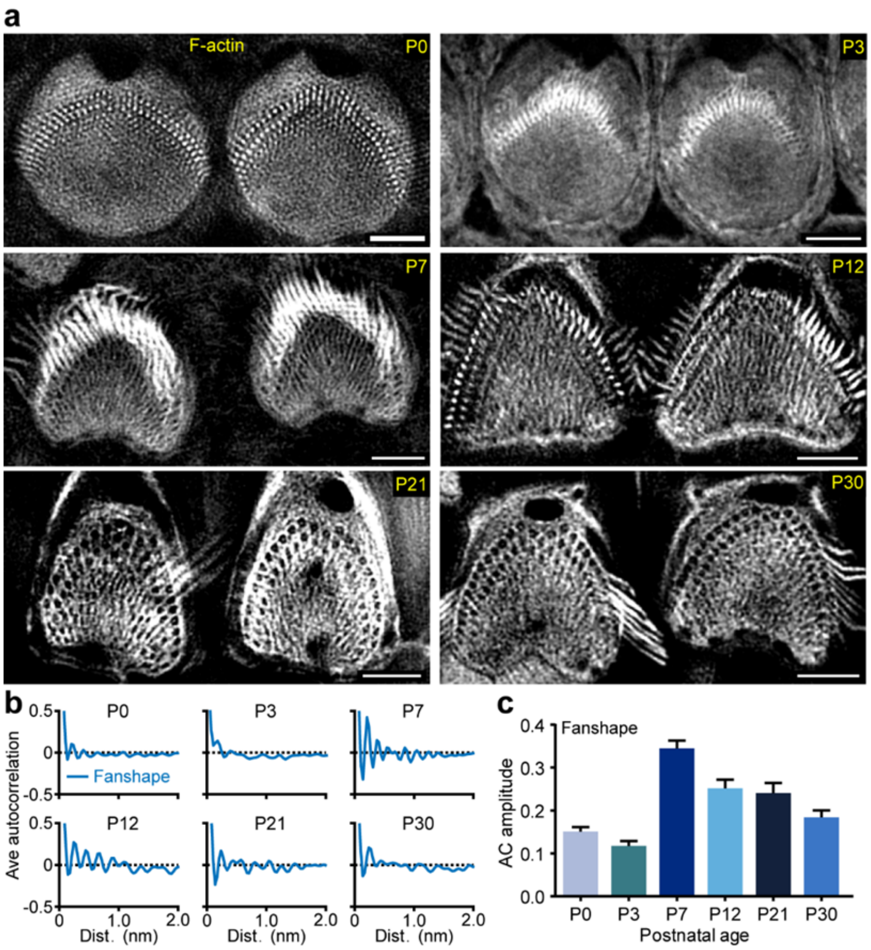

Supplementary Figure S5. F-actin fan-shaped structure in the cuticular plate during postnatal development.

a, Representative SIM images of F-actin in the cuticular plates from OHCs from P0 (n = 3 mice), P3 (n = 3 mice), P7 (n = 3 mice), P12 (n = 5 mice), P21 (n = 3 mice), and P30 (n = 5 mice). **b,** The average autocorrelation analyses for F-actin fan-shaped structure from different postnatal development stages. **c,** The average amplitudes of F-actin distribution (P0, n = 29; P3, n = 46; P7, n = 28; P12, n = 37; P21, n = 29; P30, n = 22). The amplitude was measured as the half of difference between the first peak and the average of the two first valleys of the autocorrelation curve[1]. Data are represented as Mean ± SEM. Scale bars: 2 µm.

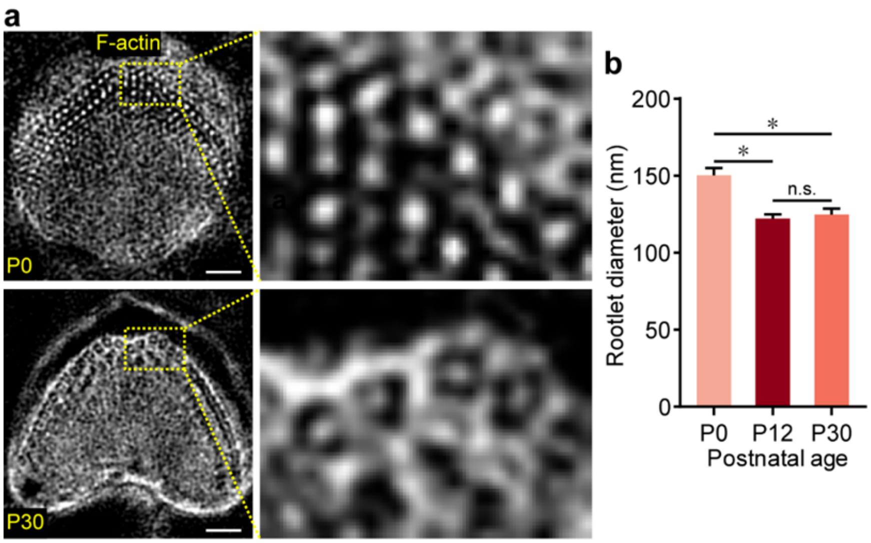

Supplementary Figure S6. The average diameter of stereocilia rootlets during development.

a, Representative SIM images of OHC with magnification of the boxed regions right from P0 and P30. The pixels of magnification images were bilinear resized for expanding 5 times to show the clearer structures of F-actin. **b,** Rootlet diameter comparison among OHCs in different postnatal development stages (P0, n = 22 rootlets; P12, n = 33 rootlets; P30, n = 21 rootlets). Data are represented as Mean ± SEM. *p<0.05 is calculated by Student's t-test. Scale bar: 1 µm.

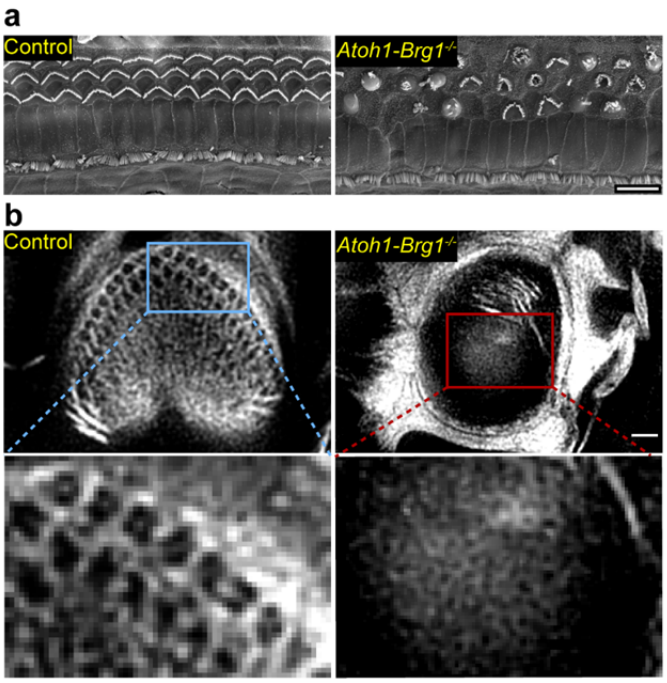

Supplementary Figure S7. Disruption of F-actin structures in hearing impaired mice.

a, SEM images of organ of corti morphology from control (n=3 mice) and *Atoh1-Brg1*$^{-/-}$ (n=3 mice) mice. **b,** SIM images and corresponding magnified regions of F-actin in OHCs from control (n=3 mice) and *Atoh1-Brg1*$^{-/-}$ (n=3 mice) mice. Scale bars: 10 µm (a), 1 µm (b).

Supplementary Movie S1. F-actin structure in cuticular plate of OHC from a P0 mouse.

Supplementary Movie S2. F-actin structure in cuticular plate of OHC from a P3 mouse.

Supplementary Movie S3. F-actin structure in cuticular plate of OHC from a P7 mouse.

Supplementary Movie S4. F-actin structure in cuticular plate of OHC from a P12 mouse.

Supplementary Movie S5. F-actin structure in cuticular plate of OHC from a P21 mouse.

Supplementary Movie S6. F-actin structure in cuticular plate of OHC from a P30 mouse.

Reference

1 Zhong, G. *et al.* Developmental mechanism of the periodic membrane skeleton in axons. *Elife* **3** (2014).
